# Co‐Designing a Community‐Based Health Literacy Programme for Individuals With Prehypertension: An Application of Ophelia (Optimise Health Literacy and Access) Process

**DOI:** 10.1111/hex.70637

**Published:** 2026-03-15

**Authors:** Pataporn Bawornthip, Jo McDonall, Decha Tamdee, Andrea Driscoll, Anastasia Hutchinson

**Affiliations:** ^1^ School of Nursing and Midwifery, Faculty of Health, Deakin University Geelong Victoria Australia; ^2^ Center for Quality and Patient Safety Research – Epworth HealthCare Partnership, Deakin University Geelong Victoria Australia; ^3^ Faculty of Nursing, Chiang Mai University Chiang Mai Thailand; ^4^ La Trobe University, School of Nursing and Midwifery Melbourne Australia; ^5^ Center for Quality and Patient Safety Research‐ Monash Health Partnership Victoria Australia

**Keywords:** co‐design, health literacy, hypertension prevention, Ophelia, participatory, rural community based

## Abstract

**Background:**

The rising prevalence of non‐communicable diseases, especially hypertension, presents a significant public health concern. Early detection and effective health promotion of hypertension remain challenging for healthcare providers. A co‐design approach was used to engage stakeholders and consumers in exploring and developing a culturally appropriate intervention. This study aimed to co‐design evidence‐based resources with local key stakeholders, focusing on hypertension prevention for individuals with varying levels of literacy and addressing barriers to behaviour change in a rural Thai community.

**Methods:**

This study was guided by the Optimising Health Literacy and Access (Ophelia) framework, specifically steps 3 to 5, to codesign a hypertension prevention programme. Participants were recruited through purposive sampling, based on recommendations from the health service advisory group. A total of 13 individuals – comprising consumers, healthcare providers, and community leaders – were invited to participate in a codesign workshop. Data were analysed using descriptive and content analysis.

**Results:**

Thirteen workshop participants generated action ideas using Problem‐Tree Analysis and the Rose, Thorn, Bud technique. These ideas were then prioritised using an Impact–Effort Matrix to determine feasible next steps. Interventions identified in the “quick wins” (do now) quadrant were selected for further development. The final health literacy interventions included: (1) a community‐based educational project on hypertension; (2) a blood pressure monitoring system for individuals with prehypertension; and (3) public relations activities to raise community awareness.

**Conclusion:**

Co‐design approaches underpinned by the Ophelia framework enable the development of tailored, culturally appropriate health literacy interventions that improve health outcomes and access to services by addressing the diverse, real‐world needs of rural communities.

**Patient or Public Contribution:**

The co‐design process involved engaging community leaders, health care providers, individuals with hypertension or prehypertension, and their family members throughout the research process.

## 1 Introduction

Worldwide, there is a rising prevalence of chronic non‐communicable disease, particularly cardiovascular disease in low‐ and middle‐income countries [1, 2]. Despite numerous initiatives aimed at promoting behaviour change and addressing lifestyle factors driving the emerging tsunami of chronic disease, achieving sustained behaviour change remains a significant challenge [3, 4]. There is increasing interest in evaluating whether using participatory approaches to the design and implementation of health promotion programmes may result in more successful behaviour change [5, 6]. In this study, a co‐design approach was used to develop a programme that aimed to improve cardiovascular outcomes for a rural population in northern Thailand, focusing on strategies to improve community‐based initiatives to support people to achieve healthy blood pressure control.

Early detection and prevention of hypertension poses challenges for primary and community care providers in Southeast Asia; however, without effective health promotion programmes, the population may experience significant socioeconomic and health consequences [1]. Key risk factors for developing hypertension include high salt intake, low educational attainment, low socioeconomic status, being overweight, alcohol consumption, smoking, and physical inactivity [1, 7]. Notably, a systematic review conducted in the Southeast Asian context by Bawornthip et al. [8] suggested that optimising blood pressure control amongst individuals with prehypertension requires encouraging the consumption of a healthy diet, restricting sodium intake, and increasing potassium intake. In remote areas, health promotion interventions for individuals with prehypertension are more likely to be effective and sustainable when developed through co‐design with community participants, ensuring the activities are appropriate and relevant to the end users [9].

Co‐design is a participatory approach that actively engages relevant stakeholders and end users throughout the development of healthcare services and health promotion initiatives, developing upon their lived experiences and practical knowledge [9, 10]. A previous meta‐analysis reported that the codesign process can enhance health behaviours and outcomes, including improvements in physical health, uptake of health‐promoting behaviours, self‐efficacy, and access to health services [11]. Several studies conducted in Australia, the United Kingdom, China, Thailand, the Philippines, and Egypt have employed codesign approaches to improve health literacy and the management of cardiovascular disease in both high‐ and low–middle‐income countries. These studies were guided by various co‐design frameworks, including the Double Diamond model, the Guidance for Reporting Involvement of Patients and the Public 2 (GRIPP2), the Hasso Plattner Institute of Design at Stanford University, and the Optimising Health Literacy and Access (Ophelia) framework [10, 12, 13, 14, 15, 16, 17, 18, 19].

The Ophelia process employs a co‐design approach to co‐create solutions aimed at improving access, promoting equity, and enhancing health outcomes by addressing health literacy needs, especially in primary health care [13, 20]. This process is guided by eight key principles: a focus on improving health and well‐being outcomes; a commitment to equity; the integration of local wisdom; responsiveness to local health literacy needs; active engagement of all relevant stakeholders in the co‐design process; adaptability to varying and evolving health literacy needs; applicability across all levels of the health system; and a focus on achieving sustainable changes in environments, practices, cultures, and policies [9]. To date, the Ophelia process has not been implemented in Thailand to support the development of health literacy for the prevention of hypertension.

Health literacy encompasses the personal knowledge and competencies developed through daily activities, social interactions, and intergenerational experiences, which are shaped by organisational structures and available resources that enable individuals to access, understand, appraise, and apply information and services to promote and maintain health and well‐being for themselves and others [9, 21, 22]. Health literacy not only focuses on access to health information but also involves the ability to critically evaluate and apply the information provided, to communicate with health care personnel and navigate the health care system [21]. Previous studies have shown that low health literacy is associated with decreased uptake of preventative and health‐promoting behaviours in individuals with prehypertension [23, 24, 25]. A survey of participants general health literacy in four regional health centres found that most Thai people had fair health literacy, and one quarter of adult group had low health literacy [26]. A previous survey conducted in a Thai rural community revealed a controversy: although the majority (90.6%) of individuals with prehypertension reported good or very good levels of health literacy, only 61.5% engaged in regular physical activity and 66.8% adhered to a healthy diet [27]. To develop effective interventions that address key barriers to successful behaviour change, it is essential to codesign interventions with consumers, health care providers and community leaders that are accessible, sustainable, and relevant [27].

The aim of this study was to co‐design with local key stakeholders’ evidence‐based resources focusing on hypertension prevention that are culturally appropriate, accessible to individuals with varying levels of literacy and address barriers to behaviour change in a Thai rural community.

## 2 Methods

### 2.1 Study Design

**Figure 1:**
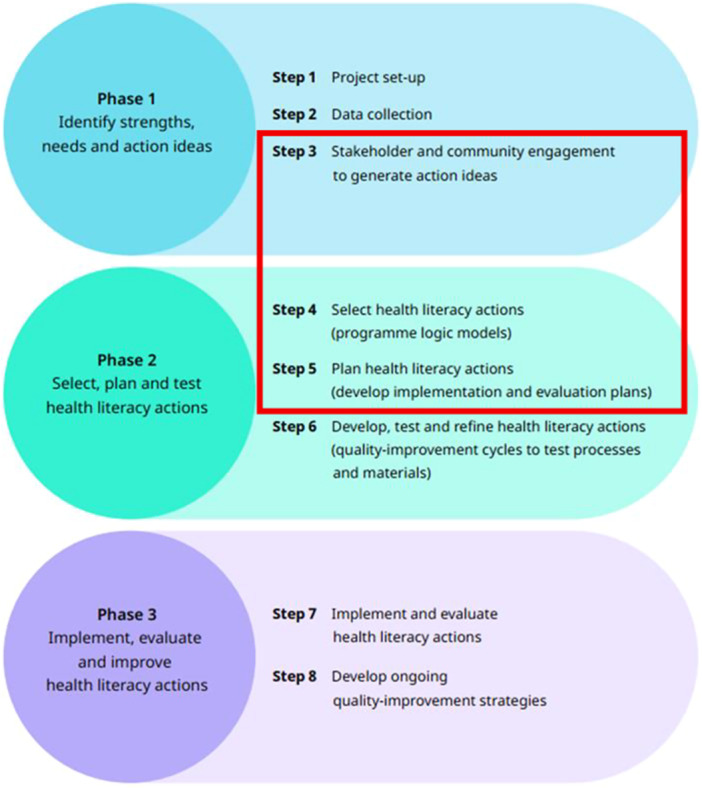
Overview of the Ophelia approach (adapted from Osborne et al., 2021).

**Figure 2:**
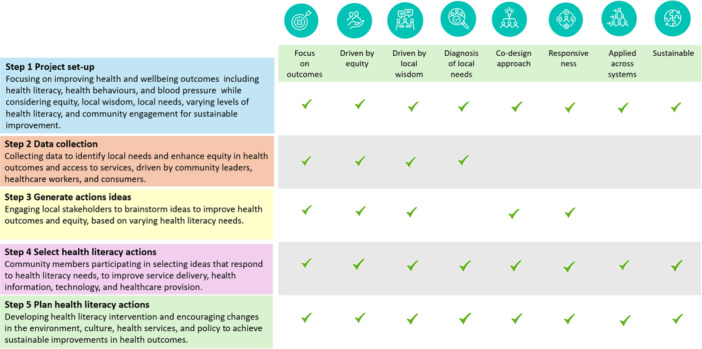
Steps of the Ophelia process with integrated principles.

Underpinned by the eight principles of Ophelia Framework, a co‐design approach was used to develop a hypertension prevention intervention (Figures 1 and 2) [9, 28]. This study is a component of a prospective multi‐phase study, Phase 1, step 1– Project step‐up involved establishment of a project advisory group in July 2024 who were involved in planning for each stage of the project.

In step 2‐Data collection was conducted in the study community to obtain an understanding of focus areas to improve health and wellbeing in the community. A cross‐sectional survey was conducted to evaluate individuals with prehypertension and their family's health literacy and participation in health promoting behaviours [27]. Focus groups were conducted with community leaders, health care workers, family members, and individuals with hypertension and prehypertension to obtain local insights about the barriers and facilitators to effective behaviour change. The purpose of this step was to identify focus areas for further intervention to overcome personal, cultural and system barriers to uptake of healthy lifestyle recommendations and to improve equity in access to primary health care, and to improve blood pressure control in the whole community.

In this article, the processes and outcomes of the following steps within phases 1 and 2 of the Ophelia Framework (Figure 1) are reported: step 3 stakeholder and community engagement to generate action ideas, step 4 select health literacy actions, and step 5 plan health literacy actions. Step 6, Develop, test, and refine health literacy actions, is ongoing and will involve an evaluation of the acceptability and feasibility of the health literacy interventions before implementation in Phase 3.

### 2.2 Setting

The co‐design study was conducted in a primary health care setting in northern Thailand, specifically in a rural community located in the Ta‐Kwang Subdistrict, Saraphi District, Chiang Mai Province [29]. The primary healthcare centre in the Ta‐Kwang sub‐district provides holistic health care services to approximately 2700 residents across seven villages. It is located about 20 kilometres (a 30‐min drive) from the central city of Chiang Mai Province. Residents typically travel between villages by bicycle or motorcycle, covering an average distance of 1–3 km. The travel from any village to the healthcare centre takes about 3–5 min by vehicle [29]. There are 1,142 households, with 1,270 males and 1,479 females. Most of the population is aged between 18 and 60 years, and most people work in agriculture or general labour.

In 2024–2025, the prevalence of hypertension and prehypertension increased from 46.3% to 49.4% and from 1.4% to 5.6%, respectively [30]. This community has a stable, close‐knit population, and there is a close and equitable relationship between the community leaders, healthcare workers and consumers who were participants in this study.

### 2.3 Ethical Considerations

This study was approved from the Deakin University Human Research Ethics Committee (2024‐129) and the Faculty of Nursing, Chiang Mai University (2024‐EXP059). Written informed consent was obtained from all participants before data collection, including permission to collect data, take photographs, and publicly present the photographs.

### 2.4 Participants and Recruitment

Participants were selected through purposive sampling based on recommendations from the advisory group. Thirteen individuals, representing consumers, healthcare providers, and community leaders, were invited to take part in the codesign workshop. The sample was drawn from the same population as in Phase 1, and invitations were extended via letter and phone call. Participant inclusion criteria were: being able to speak, communicate, read, and write in the Thai language, previous participation in Ophelia Phase 1 (step 2: data collection) and willing to provide informed consent for study participation.

### 2.5 Data Analysis

For this study, field notes and written information on sticky notes were collected. The data was analysed using descriptive and content analysis. The conceptual content analysis was conducted using word counts and keyword‐in‐context (KWIC) analysis [31, 32]. KWIC is a technique used to assess the context in which keywords appear by examining the surrounding words or sentences, following an analysis of word frequency to gain a deeper understanding of their contextual meaning [31].

### 2.6 Study Procedures

#### 2.6.1 Step 1 Project Set‐Up

Researchers established a project team and an advisory group comprising the head of a health‐promoting hospital and staff from Ta‐Kwang municipality along with the project's time frame and budget. The advisory group consisted of four participants: the head of the health‐promoting hospital, a nurse practitioner, a village health volunteer, and a community leader. The head of the health‐promoting hospital was responsible for recommending key stakeholders to participate in workshops and project planning. The nurse practitioner was responsible for recruiting and contacting participants, as well as preparing the meeting room. All members of the advisory group shared responsibility for approving interventions during group meetings and providing suggestions that were appropriate for the community.

#### 2.6.2 Step 2 Data Collection and Extraction

A cross‐sectional survey was conducted to assess health literacy levels amongst individuals with prehypertension [27]. Additionally, focus groups with key community stakeholders were conducted to identify local problems, strengths, needs, and barriers for people in their community who are at risk of hypertension. Three focus groups were conducted (*n* = 27). There were 21 females and 6 males. Participants included community leaders (*n* = 9) and healthcare providers (*n* = 10). Consumers (*n* = 8) included individuals with prehypertension or hypertension and their family members. From the focus group discussions, four themes were identified using the Health Belief Model as a framework and are presented as follows: (1) the rising prevalence of hypertension and its social consequences; (2) hypertension as an asymptomatic condition; (3) contextual and cultural barriers; (4) enhancing enablers for behaviour change.

The lead researcher then developed vignettes based on the results from the survey and focus groups and sent them to the advisory group for confirmation that these vignettes captured the key concepts that emerged from the focus group discussions and that the vignettes were easy to understand.

#### 2.6.3 Step 3 Co‐Design – Participants and Process for Codesign Day

The co‐design workshop was held in the meeting room at Bankwae Health Promoting Hospital. Thirteen participants participated, including one deputy mayor of Ta‐Kwang municipality, two village leaders, the head of Bankwae Health Promoting Hospital, a nurse practitioner, four village health volunteers, two people with prehypertension, one person with hypertension, one caregiver or family member who cares for people with prehypertension and hypertension. The lead researcher (PB) and assistant researchers took photographs during the workshop, recorded field notes, and collected sticky notes for documentation.

##### 2.6.3.1 What the Codesign Activities Were

**Figure 3:**
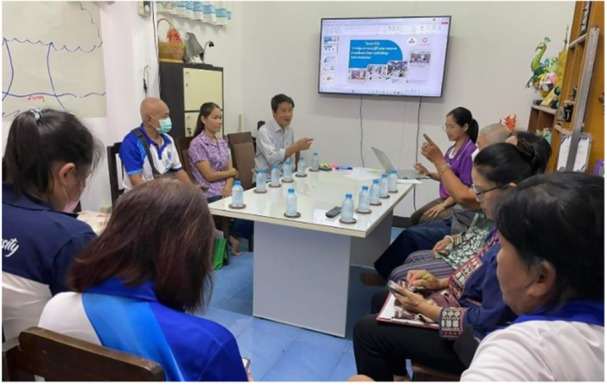
The lead researcher presented workshop objectives and the results.

**Setting the Scene:** At the beginning of the co‐design workshop, the researcher (PB) presented workshop objectives and shared the results from Ophelia step 2 (focus groups and cross‐sectional survey) [27] to workshop participants, giving them an overview of the understanding individuals with prehypertension have regarding the importance of maintaining healthy blood pressure levels and the barriers faced by individuals in making healthy lifestyle choices (Figure 3).

The researcher (PB) created and presented them via PowerPoint presentations vignettes in a lay summary format for stakeholders and community members to help them understand how a person with a specific level of health literacy would navigate the health system and understand relevant health information. Workshop participants (local stakeholders) confirmed the information and engaged in a brainstorming session to generate potential solutions to address these problems.

During the co‐design workshop techniques from the LUMA Institute, such as Problem‐Tree analysis to guide problem‐solving direction, and Rose, Thorn, Bud to identify issues and insights [33] were applied. These brainstorming and problem‐solving activities are designed to promote equitable interaction between all workshop participants, and to overcome pre‐existing hierarchies. These techniques enabled equitable participation in the co‐design workshop in alignment with the principles of the Ophelia process.

**Problem‐Tree Analysis:** The researcher encouraged participants to frame the problem in their local community by using Problem Tree Analysis to discuss and explore the causes and effects of a particular issue. This helped participants better understand the chain of connected circumstances that led to the current situation. The Problem Tree Analysis presented the causes (roots) from the effects (branches) of a central issue (trunk) [33].

**Rose Thorn Bud:** The researcher used the ‘Rose, Thorn, Bud’ technique to encourage local stakeholders to identify positive experiences, negative experiences, and new goals that are potentially related to the prevention of hypertension for individuals with prehypertension [33]. The participants were separated into two groups. Each group was asked to generate ideas related to each concept and record one idea per sticky note, using different colours for each category. Workshop participants identified positive aspects (Roses), such as previously successful interventions or projects conducted in their community, which were presented on pink sticky notes. For negative experiences or challenges (Thorns), local stakeholders were asked to consider barriers to engaging in health‐promoting behaviours such as making healthier food choices, increasing physical activity, maintaining a healthy weight, and monitoring blood pressure with these ideas recorded on blue sticky notes. Participants were encouraged to develop the prevention and management of hypertension in a culturally appropriate way and accessible to individuals with varying literacy levels (Buds). The workshop participants generated ideas about how to reduce these barriers. Additionally, the ideas were categorised into four levels: individual, family, practitioner and policy levels. The researcher started the conversation with prompts such as: “How might we…?” questions and encouraged participants to generate ideas and write these on green sticky notes. Each group presented and stuck their ideas on the meeting room wall. The pool of ideas generated from step 3 was used to set priorities for action and design interventions appropriate for the local problems identified.

#### 2.6.4 Step 4 Select Health Literacy Actions

**Figure 4:**
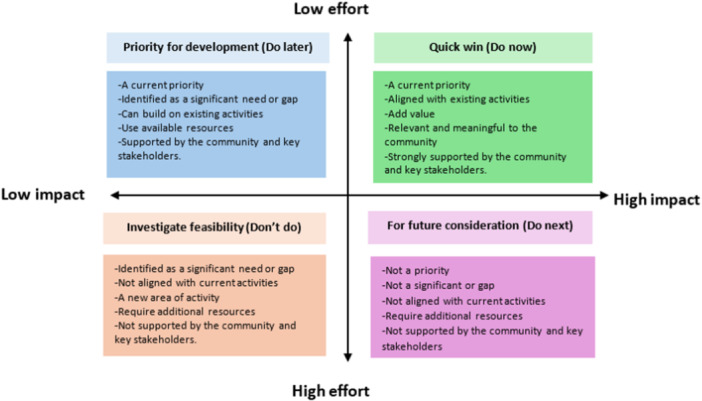
An impact effect prioritisation matrix.

As the co‐design workshop progressed, local stakeholders used the impact effort matrix to prioritise ideas for designing health literacy interventions (Figure 4). The matrix categorises ideas along two axes: effort to implement (ranging from easy to hard) and potential impact (ranging from low to high) [9, 33, 34]. Based on these dimensions, all ideas were sorted into four categories:

Category 1: Quick Wins (Do Now)

These interventions are current priorities, aligned with existing activities, add clear value, are relevant and meaningful to the community, and are strongly supported by both the community and key stakeholders.

Category 2: Priority for Development (Do Later)

These ideas represent current priorities and address significant needs or gaps. They can build on existing activities, utilise available resources, and are supported by the community and key stakeholders, but may require further planning or coordination.

Category 3: Investigate Feasibility (Don't Do)

These ideas relate to areas of potential need but are not aligned with current activities. They often represent new areas of work, require additional resources, or lack sufficient support from the community and key stakeholders. As such, they require further investigation before implementation.

Category 4: For Future Consideration (Do Next)

These interventions are not immediate priorities, do not address significant gaps, are not aligned with current initiatives, and may lack community or stakeholder support. They may also require substantial new resources. These ideas are set aside for possible future development.

#### 2.6.5 Step 5 Plan Health Literacy Actions

The purpose of this step was to determine who would implement the health literacy interventions, along with the required resources and materials. The implementation and evaluation plan were co‐designed with key stakeholders who demonstrated support for and understanding of the health literacy interventions. This co‐design process involved detailing the health literacy actions, including their objectives, relevant health literacy domains, duration and location, specific processes, required materials, organisational responsibilities, evaluation plan, and anticipated short‐term and long‐term outcomes.

## 3 Findings

### 3.1 Characteristics

The characteristics of the 13 participants involved in the co‐design activities are presented in Table 1. Community leaders included a mayor, subdistrict leaders, and village leaders. Healthcare workers included senior health‐promoting hospital management, nurses, and village health volunteers. The lead researcher (PB) and two research assistants were present at the workshop to facilitate and take notes of all workshop activities. All participants were fully engaged throughout the duration of the workshop.

**Table 1:** Characteristics of workshop participants (*n* = 13).

Characteristics	*n*
Sex	
Female	10
Age (years) Mean (SD) 52.44 (5.66) Range 36–73	
Age group	
30–40	1
41–50	2
51–60	3
61 and over	7
Education	
Primary education	3
High school	5
Bachelor's degree or over	5
Role/position	
Community leaders	3
Healthcare workers	6
Individuals with hypertension	1
Individuals with prehypertension	2
Family member	1

### 3.2 Generate Action Ideas (Step 3)

Local stakeholders participated in the co‐design workshop to develop potential actions or interventions aimed at: 1) improving knowledge about the importance of maintaining blood pressure within a healthy range, 2) increasing health literacy skills related maintaining blood pressure in a healthy range, and 3) addressing barriers to successful behaviour change and uptake of healthier lifestyle behaviours.

#### 3.2.1 Problem‐Tree Analysis

The central issue identified from this activity was that the number of people with hypertension and stroke was increasing in their community. Participants identified that this was related to low self‐awareness of poorly controlled blood pressure and unhealthy lifestyle behaviours such as low exercise participation and consumption of a high salt diet. The effects include patients being at risk of cardio‐ and cerebrovascular complications, surging demand for primary care services, a shortage of staff and insufficient budget to provide health promotion, screening and treatment of hypertension.

#### 3.2.2 Rose Thorn Bud Activity

**Figure 5:**
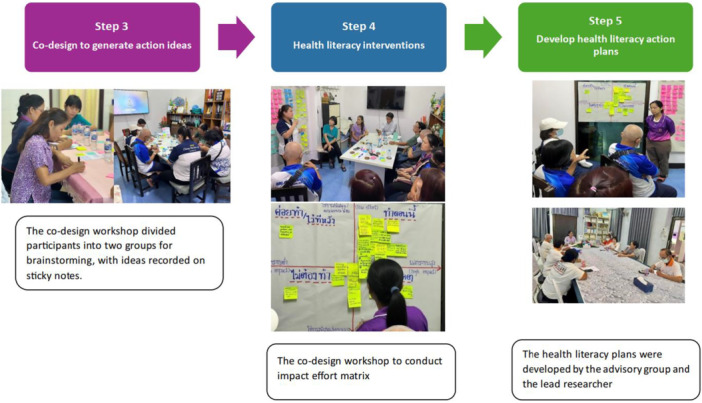
The co‐design process to develop health literacy interventions.

After completing the first co‐design activity, the researcher divided the workshop participants into two groups to encourage conversation, interaction, and brainstorming using the Rose, Thorn, Bud technique (Figure 5). Participants wrote their ideas on different coloured sticky notes and presented them within their groups (Table 2).

**Table 2:** The results of Rose, Thorn, Bud codesign activity.

Rose	Thorn	Bud
1. Annual health check 2. Measuring the blood pressure of village health volunteers in various activities [2] 3. After screening risk groups, some people realise, and some people are the same. 4. Designing a blood pressure measurement service in every village to screen out risk groups. 5. Meeting with a group to share ideas 6. People who believe in a concept and apply it in their daily lives often become advocates for that concept and role models for others. 7. A public announcement made over the village loudspeaker system by the village head or sub‐district head. 8. Set a time frame for the activity within 3 months. 9. No Alcohol, Soda, or Beer Project 10. There used to be a health market in 2017. 11. Chemical‐free Vegetable Growing Group. 12. A campaign to reduce sugar, fat, and salt. 13. Reduce foods that increase the risk of high blood pressure, such as fatty foods, alcohol, cigarettes, and salty, fatty and sugary foods. (Previously, I consumed a lot of sweets, but my intake has been reduced.) [6] 14. Determine appropriate portion sizes for each meal to maintain a healthy diet [2]. 15. Consume a balanced diet of all 5 food groups [5]. 16. Reducing the use of MSG and seasonings improves health, promotes better sleep, reduces stress, aids in weight loss, improves skin, and decreases the need for pain medication. 17. Exercise [9] 18. Do exercise every day by cycling every evening. 19. Don't stress, relax your mind [3]. 20. Have enough rest [3]. 21. Find something to do in my free time. 22. Strengthen family immunity. 23. Growing flowers at home makes the house beautiful and improves both mental and physical health. 24. Taking care of one's health	1. Working people claim they don't have time. 2. Eat out 3. Working people often opt for ready‐made food due to time constraints. 4. Unable to avoid unhealthy food 5. An inadequate level of education leads to ignorance of sodium intake. 6. The local cuisine often includes foods high in sodium, which can contribute to high blood pressure. 7. Still consuming sweet, savoury, and salty foods. 8. I find bland food unappetising, causing me to eat less, feel stressed, and have trouble sleeping [2]. 9. Some people don't want to spend a lot of money buying new seasonings because they think it's a waste of money. 10. Not exercising regularly. 11. Some people believe that eating low‐sodium food makes food taste bad. 12. Lately, I haven't been exercising. I don't have time. I go to social events often [3]. 13. People who have problems will say, ‘Never mind.’ 14. I don't have time. 15. People couldn't exercise due to COVID‐19. 16. People are unaware and think they can get free treatment if they have high blood pressure.	1. Set up an exercise club. and places to exercise such as aerobic dance club. 2. Provide funding for training of relevant agencies. 3. Create a food club to cook with reduced sodium. 4. Process local herbs instead of seasonings. 5. Educate the general public in the community about high blood pressure. 6. There are many different forms of media available to change behaviour. 7. Training to be provided on the topic of eating. 8. The current budget is insufficient to cover the entire population; additional funds are needed to support the promotional project. 9. Training should be held twice a year. 10. Cooking demonstration with sodium levels shown. 11. Organise a low‐sodium food club. 12. To promote good physical and mental health, and a healthy community environment. 13. To build a network and distribute information to villagers about hypertension. 14. A model of each seasoning to know the amount of sodium. 15. Patients with stroke share their symptoms and experiences. 16. Emphasise the policy of reducing sugary drinks. 17. All community organisations are working together to raise awareness such as developing a monitoring system. 18. Continuous public relations. 19. Provide information to motivate both exercise and healthy eating. 20. Create a Group line for people at risk of hypertension. 21. Developing motivational activities for behaviour modification. 22. Continuous financial support for ongoing activities. 23. The health centre buys exercise equipment. 24. Fast and efficient healthcare services with consulting. 25. Monitor blood pressure weekly. 26. People who are already maintaining their health. 27. Modern fitness facility in our sub‐district. 28. Health consultations are available from doctors. 29. Let's work together to raise awareness about diet and lifestyle among people at risk of high blood pressure.

### 3.3 Health Literacy Interventions (Step 4)

Workshop participants collaborated in this step, using outcomes from Step 3 to select health literacy actions that could be implemented to improve health literacy and health behaviours and improve blood pressure control. At the beginning, the researcher confirmed the project focus and scope and aimed to specify the expected outcomes, including short‐term, medium‐term and long‐term outcomes. The short‐term outcomes included: (1) enhancing knowledge and health literacy and (2) increasing self‐awareness amongst individuals with prehypertension. The medium‐term outcomes focused on: (1) individuals with prehypertension adopting improved health behaviours and achieving blood pressure control, and (2) the population in the community concerned in hypertension prevention. The long‐term outcomes were as follows: (1) the population in Ta‐kwang subdistrict, including children, working‐age groups, and aging groups, attaining good health without hypertension, (2) the Ta‐Kwang community becoming free from hypertension and at‐risk groups, and (3) reducing the number of individuals with prehypertension.

Then, workshop participants prioritised the ideas by using an impact effort matrix to progress next. The ideas in the quick wins (do now) area were voted to be potential interventions, and these interventions or innovations were developed into phototypes, considering desirability, viability, and feasibility criteria (Figure 5). The results from the impact–effort matrix informed strategies to encourage dietary behaviour change, such as cooking demonstrations focused on reducing sodium and incorporating local herbs. Additional strategies included promoting physical activity, delivering health education through multiple media channels, sharing information via social media, and raising awareness among community residents (Table 3).

**Table 3:** The result from the impact effort matrix.

Do now	Do later	Do next	Don't do
Decreasing sodium intake 1. Cooking demonstration with low sodium levels shown [2]. 2. Process local herbs instead of high salt seasonings. 3. A model of each seasoning to know the amount of sodium. Community‐awareness raising 1. Educate the general public in the community about high blood pressure. 2. Should have many different forms of media available to change behaviour. 3. Training should be held twice a year. Community engagement and a whole of community approach 1. Let's work together to raise awareness about diet and lifestyle among people at risk of high blood pressure. 2. To promote good physical and mental health, and a healthy community environment. 3. To build a network and distribute information to villagers about hypertension. 4. Continuous public relations. Consumer‐led and establish consumer networks 1. Patients with stroke share their symptoms and experiences. 2. Provide information to motivate both exercise and healthy eating [2]. 3. Create a Group line for people at risk of hypertension. 4. Developing motivational activities for behaviour modification. Collaboration between providers 1. All community organisations are working together to raise awareness such as developing a monitoring system. Funding 1. Provide funding for training of relevant agencies. 2. The current budget is insufficient to cover the entire population; additional funds are needed to support the promotion project.	1. Set up an exercise club and places to exercise such as the aerobic dance club. 2. The health centre buys exercise equipment.	1. Modern fitness facility in our sub‐district. 2. Health consultations are available from doctors. 3. Fast and efficient healthcare services with consulting. 4. Organise a low‐sodium food club. 5. Emphasise the policy of reducing sugary drinks. 6. Continuous financial support for ongoing activities. 7. The current budget is insufficient to cover the entire population; additional funds are needed to support the promotion project.	1. Monitor blood pressure weekly.

By the end of the activity, local stakeholders co‐designed the intervention and identified responsible persons in collaboration with the lead researcher (PB). The intervention was developed based on evidence from a literature review and suggestion from the advisory group to ensure its relevance and suitability for the community [8, 34, 35, 36].

### 3.4 Develop Health Literacy Action Plans (Step 5)

**Figure 6:**
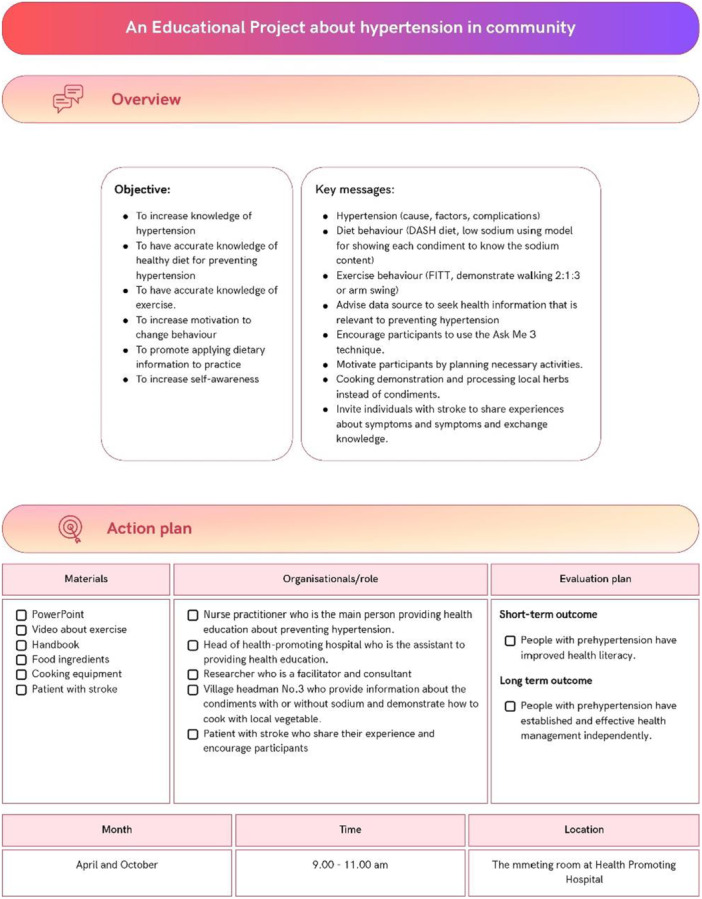
An educational project about hypertension in the community.

**Figure 7:**
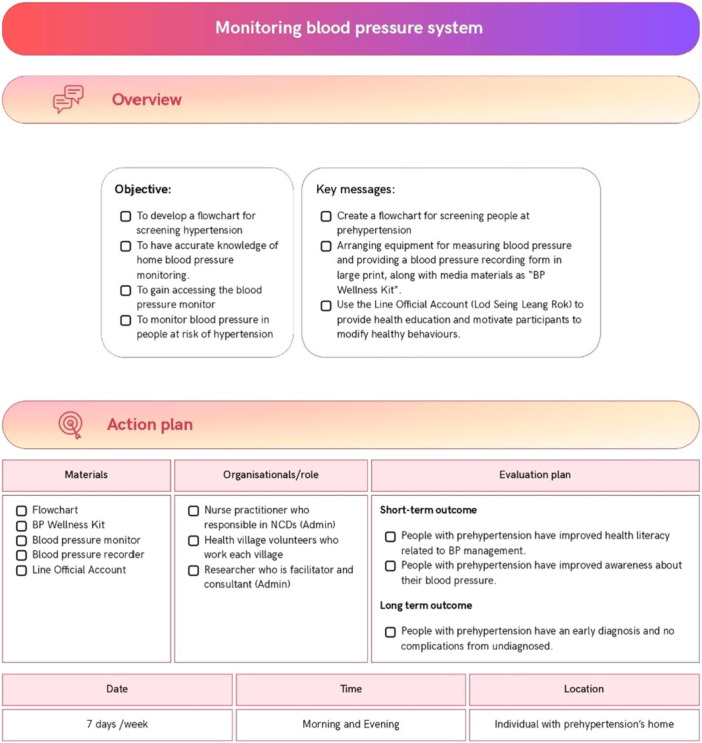
Monitoring blood pressure system for individuals with prehypertension in community.

**Figure 8:**
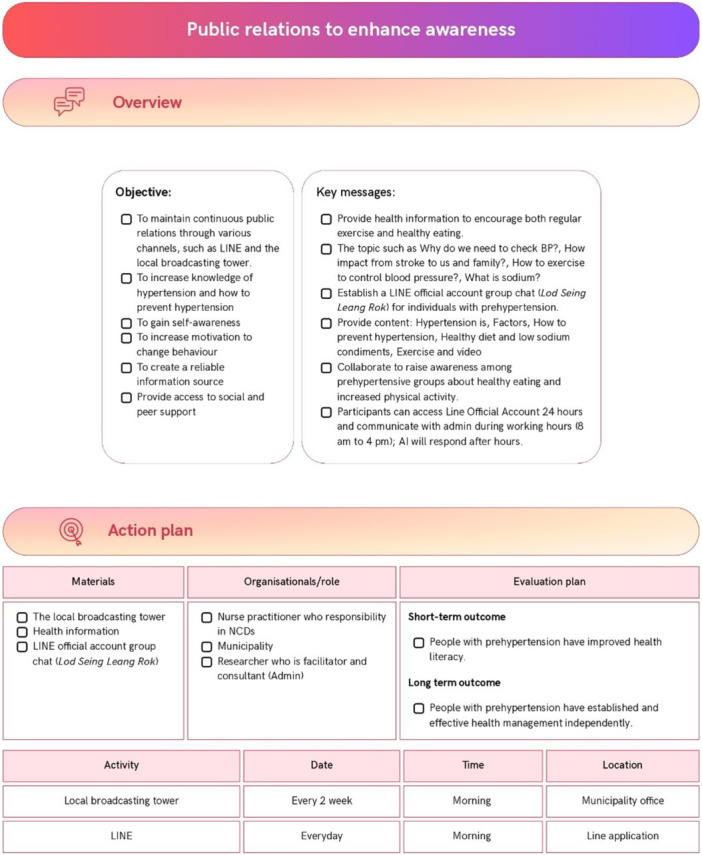
Public relations to enhance awareness.

The implementation and evaluation plans were developed by the advisory group and the lead researcher (PB) (Figure 5). These plans include a community‐based educational project on hypertension (Figure 6), a blood pressure monitoring system for individuals with prehypertension (Figure 7), and public relations efforts to raise awareness (Figure 8). All interventions addressed components of health literacy, including the ability to access health information and services, understand health information, make informed decisions regarding health information and services, and apply health information and services in daily life.

## 4 Discussion

This study utilised co‐design with local stakeholders to develop a health literacy intervention, underpinned by the Ophelia framework, to improve health outcomes for individuals with prehypertension in a rural community and to enhance access to health promotion services. Health literacy interventions developed by community leaders, healthcare providers, and consumers were tailored and culturally appropriate to meet the needs of end users. The co‐design approach addresses a significant gap in designing effective healthcare delivery for hypertension prevention by incorporating community‐driven strategies such as using local herbs instead of monosodium glutamate, creating social media groups to share information, promoting public awareness about hypertension and healthy behaviours, and improving blood pressure monitoring systems.

To date, only one study has used a co‐design process to develop a chronic care model in urban Thailand [12]. Koontalay et al. (2024), used a design thinking model proposed by Stanford University to create a nurse‐led case management service for patients with heart failure, supported by a multidisciplinary team. This study is the first to use a co‐design approach to improve health literacy for hypertension prevention in a Thai rural community. Although the target groups were different, the co‐design approach was shown to enable active and meaningful engagement with healthcare workers, end users, and organisational or community leaders throughout the process of designing health services and health promotion activities, drawing on their experiences and practical knowledge [9, 12]. Moreover, our study included a local advisory group to support the research process by identifying key community members who could provide valuable information and insights into the real situation within the community. Previous study has suggested that a stakeholder advisory panel is useful for gaining richer insights from those with lived experience of using and working within health services [10].

In the co‐design process, researchers used the Rose, Thorn, Bud technique to encourage participants to generate ideas, which were written on sticky notes in different colours. This approach was useful for identifying and reflecting on emerging patterns later on [33, 37]. Additionally, participants were allowed to write anything on sticky notes without including their names, which helped them express their ideas freely without fear or concern about others in the group [10]. To select the health literacy intervention, we applied an impact–effort matrix to prioritise activities, encouraging participants to consider which actions could be successfully implemented with low effort. The prioritisation matrix is a decision‐making tool used to support communication and guide the selection of target strategies to address issues [38, 39].

Including a diverse participant group in the co‐design workshop, we enabled better coordination and communication of issues, particularly those related to the aggregated group or organisation. For example, we communicated the funding and financial challenges to the municipality and the head of the health‐promoting hospital, to inform them and encourage consideration of additional funding for the health promotion project. In terms of promoting reduced use of condiments with high sodium content, we engaged a community leader with local wisdom and experience, who volunteered to demonstrate how to cook using local herbs (Chaya). A previous study confirmed that chaya leaves provide a good aroma and umami taste, comparable to natural seasoning powder or monosodium glutamate [40].

To enhance self‐awareness and motivation in adopting health behaviours, interventions should address the individual, family, and community levels. Health education was also one of the interventions requested by participants; however, it should include more active participation and interactive sessions, such as cooking demonstrations and guidance on home‐based exercises. Educational media should be provided in various formats – including books, brochures, videos, photos, and audio – through multiple channels to enhance both effectiveness and accessibility [17, 18, 41, 42]. This study used municipal and village broadcasting towers to deliver health information in the local language (Thai Lanna) during the mornings, which was suitable for most of the population. This finding is consistent with previous studies indicating that the use of local media for health communication in Thai rural areas is effective, as community members who are involved in producing the media tend to feel a sense of ownership [43]. Moreover, health information that was valid, credible, easy to understand, and applicable to daily life was provided to individuals with prehypertension via social media, specifically through the Line application.

### 4.1 Strengths and Limitations

By using the Ophelia Framework to guide the development of this research project, the researchers incorporated structured activities to capture local wisdom and develop a proposed intervention that addressed the varying health literacy needs of the community and focused on improving health outcomes. Second, the ideas were generated from the diverse perspectives of local key stakeholders, including community leaders, healthcare providers, and consumers, which contributed to the development of a health literacy intervention that was both tailored and culturally appropriate for the community.

Some limitations were identified and considered. Firstly, this study was conducted in a single rural community using a co‐design approach, which may limit the transferability and generalisability of the findings to other settings. However, future studies can apply the co‐design approach to develop interventions aimed at improving health outcomes and promoting equity in health services. Secondly, all participants were recruited from literate populations, which may have excluded individuals who are illiterate but still have valuable experiences to share. Future studies could include vulnerable groups by allowing them to express their experiences through drawing or by involving their family members in the process. Thirdly, as most participants were female, the study may have missed information related to male perspectives. Future studies should recruit participants with greater attention to gender balance. Finally, due to the small size of this community all the participants in the co‐design workshop knew each other well (despite differences in their roles in the community), this decreased some of the formal hierarchical relationships that occur in larger communities, and this enabled equitable participation in the co‐design activities used. The transferability of these findings to larger, more formal and hierarchical settings in Thailand is unknown.

Future research should conduct a pilot study to assess the feasibility, acceptability, and accessibility of the intervention.

## 5 Conclusion

Using a co‐design approach underpinned by the Ophelia framework can support the development of health literacy interventions aimed at improving health outcomes and access to health services. People living in rural communities have diverse health literacy needs; therefore, interventions should be grounded in the real‐life context of the community. Tailored and culturally appropriate interventions based on co‐design can help bridge existing gaps and motivate healthy behaviours. Not only can individuals with high health literacy adopt healthy behaviours, but those with lower health literacy levels can also be motivated to make positive changes when supported appropriately [27].

## Author Contributions

**Pataporn Bawornthip:** conceptualization, formal analysis, investigation, methodology, writing – original draft, review and editing. **Jo McDonall:** validation, writing – original draft, review and editing. **Decha Tamdee:** validation, writing – original draft, review and editing. **Andrea Driscoll:** conceptualization, methodology, validation, writing – original draft, review and editing. **Anastasia Hutchinson:** Conceptualization, methodology, validation, supervision, writing –original draft, review and editing.

## Funding

The authors received no specific funding for this work.

## Ethics Statement

This study was approved from the Deakin University Human Research Ethics Committee (2024‐129) and the Faculty of Nursing, Chiang Mai University (2024‐EXP059). Informed consent, including permission to collect data, take photographs, and publicly present the images, was obtained from all participants before data collection. Participants were informed that their participation was voluntary and that the questionnaires were anonymous.

## Conflicts of Interest

The authors declare no conflicts of interest.

## Data Availability

Data underpinning the findings of this study can be obtained from the corresponding author upon reasonable request.
